# PCR-confirmed malaria among children presenting with a decreased level of consciousness in Angola: a prospective, observational study

**DOI:** 10.1186/s12936-023-04556-9

**Published:** 2023-04-22

**Authors:** Okko Savonius, Cintia F. de Souza, Cláudia Fançony, Manuel Leite Cruzeiro, Miguel Brito, Tuula Pelkonen

**Affiliations:** 1 New Children’s Hospital, Pediatric Research Center, University of Helsinki and Helsinki University Hospital, Stenbäckinkatu 9, 00290 Helsinki, Finland; 2 Health and Technology Research Center, Escola Superior de Tecnologia da Saúde de Lisboa, Instituto Politécnico de Lisboa, Lisbon, Portugal; 3 Instituto Oswaldo Cruz, Fundação Oswaldo Cruz (IOC-Fiocruz), Rio de Janeiro, Brazil; 4 Centro de Investigação em Saúde de Angola (CISA), Caxito, Angola; 5 Hospital Pediátrico David Bernardino, Luanda, Angola

## Abstract

**Background:**

In malaria-endemic areas, children presenting to hospitals with a decreased level of consciousness remain a diagnostic dilemma. The definition of cerebral malaria in a comatose child demands exclusion of other possible reasons, which requires in-depth investigations that are not easily available. The aim of this study was to investigate the frequency and clinical characteristics of PCR-confirmed malaria in a cohort of children with a decreased level of consciousness, look for potential features that would aid in differentiating children with malaria from those without, and assess the performance of traditional thick film microscopy against the cytb-qPCR-method.

**Methods:**

A total of 345 children aged 30 days–15 years old, presenting to Hospital Pediátrico David Bernardino in Luanda, Angola, with a decreased level of consciousness (Glasgow coma scale score < 15) were prospectively enrolled during 2014–2017. Malaria was defined as a positive cytb-qPCR result on any occasion in hospital. The clinical course and laboratory parameters were compared between children with malaria and those without. The performance of thick film microscopy was analysed against the PCR method.

**Results:**

161 of 345 children (46.7%) had a positive malaria PCR test result. All cases were *Plasmodium falciparum* species, and 82.6% (133/161) fulfilled the WHO criteria for severe malaria. Overall, children with malaria presented to hospital with a shorter duration of symptoms and less convulsions pre-admission compared to those without malaria. The median GCS score on admission was 8, which did not differ between children with or without malaria. Clinical findings on admission were mostly similar across the whole cohort, but an infection focus outside the central nervous system was more common in malaria-negative children. Moreover, severe anaemia, thrombocytopenia, and high CRP levels occurred more frequently in children with malaria. The case fatality ratio was 28.5% (91/319) and did not differ between parasitaemic children and those without malaria, although parasitaemic children died sooner after hospital admission. When neurological sequelae were also considered, a positive malaria test was associated with a better outcome. The performance of thick film microscopy against PCR yielded a sensitivity of 96.8% and a specificity of 82.7%.

**Conclusions:**

In this cohort of children with a decreased consciousness, the frequent presence of a malarial infection could not be judged from the clinical findings on admission, but the combination of profound aneamia, thrombocytopenia, and a high CRP level increased the odds of a positive malaria test result. Mortality remained high regardless of etiology, but malaria infection associated with fewer neurological deficits at discharge. Thick film microscopy performed well compared to the cytb-qPCR method.

**Supplementary Information:**

The online version contains supplementary material available at 10.1186/s12936-023-04556-9.

## Background

Malaria accounted globally for more than 600,000 deaths in 2021, the majority of which were in children [1]. The disease remains endemic in Angola, where both the incidence and mortality due to this disease have increased during the last years [1].

Children presenting to hospital with a decreased level of consciousness remain a diagnostic dilemma in malaria-endemic areas. In a parasitaemic child, decreased consciousness might be the result of cerebral malaria (CM), but likewise a sign of bacterial meningitis, viral encephalopathy and metabolic disturbances [2]. These conditions might also coexist [2]. Moreover, other complications of severe malaria such as hypoglycemia, severe anaemia and acidosis may also impair the consciousness [2]. Thus, an accurate and prompt discrimination between malarial and non-malarial causes of a decreased level of consciousness as well as identification of treatable metabolic disturbances is essential to improve case management and reduce deaths.

CM, the most lethal form of *Plasmodium falciparum* infection, has traditionally been defined as a coma in a child with malaria parasitaemia not attributable to another cause, and occurs in roughly one of ten children seeking hospital care due to malaria [3]. The mortality rate of CM is around 20%, while the pathophysiology of this disease remains debated [4, 5]. The diagnosis of CM is challenging in endemic regions where asymptomatic parasitaemia is common, and despite that the presence of malarial retinopathy is suggestive of CM, ophthalmoscopy is seldom available in resource-poor settings [6]. Missing the diagnosis of CM has devastating consequences, but presumptive anti-malarial treatment also comes with a cost, possibly leading to over-prescription of anti-malarials and in turn to increased anti-malarial drug resistance [7, 8]. Additionally, the definition of cerebral malaria in a comatose child demands exclusion of other possible reasons, which require in-depth investigations seldom easily available.

Microscopy is considered the gold standard for detecting malaria and identifying the species in clinical settings. However, its performance has been reported to vary according to parasite density, transmission intensity and the technicians’ level of expertise, among others [9–12]. Moreover, as the parasites are usually sequestered in capillaries and venules during a severe *P. falciparum* malaria, the parasite density determined by peripheral blood slide may be inaccurate [13].

The study aims were to (1) study the frequency, clinical characteristics, and the in-hospital clinical course of malaria during 2014–2017 among a cohort of Angolan children with a decreased level of consciousness; (2) search for potential clinical and laboratory characteristics that would aid in differentiating children with malaria from those without; and (3) compare the performance of routine microscopy against the cytb-qPCR-method in detecting malaria parasitaemia in these children.

## Methods

### Study design and participants

In this prospective, observational single-centre study, the frequency and clinical characteristics of malaria were investigated in a cohort of children presenting to Hospital Pediátrico David Bernardino in Luanda with a decreased level of consciousness defined as a Glasgow coma scale (GCS) score under 15.

Hospital Pediátrico David Bernardino in Luanda is the largest paediatric referral hospital in Angola, comprising 500 beds and receiving 500 daily attendees. Patients were enrolled at the hospital’s emergency department during a 3-year period starting in October 2014 (10.10.2014–24.10.2017). The inclusion criteria were age between 30 days and 15 years old, a GCS score < 15, an obtained filter paper sample for malarial PCR analysis, and signed informed consent from the guardian. Exclusion criteria included altered consciousness caused by trauma, or a known operated hydrocephalus.

### Data collection

Clinical and sociodemographic data were prospectively collected on specifically designed structured questionnaires, and additional information was obtained from hospital records if needed.

Sociodemographic variables included age, sex, family structure, housing conditions and attendance of school or day-care. Clinical data, regarding the disease course before hospital admission, included information on previous illnesses and medications, duration and symptoms of the current illness, and potential given treatments. On arrival to hospital, the number of signs of raised intracranial pressure were registered, along with any convulsions at the emergency department, signs of dehydration or malnutrition, and potential infections outside the central nervous system (CNS) such as pneumonia or otitis media. At discharge, the attending physician completed a neurological examination in order to detect potential neurological deficits.

Blood was collected by venipuncture on admission, following the routine of the hospital. Baseline laboratory analyses were performed at the attending physician’s discretion, including hematological and biochemical determinations (blood counts, renal function tests, liver functions tests, bilirubin levels), thick blood smears for detection of malarial parasites, screening for HIV by serology, and screening for sickle cell disease by Hb solubility testing. The microscopy for detection of malaria parasites was done as usual in the hospital according to local instructions, using Giemsa-stained thick blood films. The number of parasites per microlitre of blood was counted in relation to the number of white blood cells.

As part of the study protocol, the level of serum C-reactive protein (CRP) (QuickRead go, Orion Diagnostica, Espoo, Finland) and blood haemoglobin were followed up on arrival and on day 4 (± 1 day), and more frequently if needed. Blood glucose was measured 2–4 times during the first day at the ward, and thereafter depending on the patient’s clinical condition. A follow-up measurement was taken on day 4 from all children. Moreover, a cerebrospinal fluid (CSF) sample was to be obtained from all children on admission for customary microbiological, biochemical, and cytological analysis.

In addition, a blood sample from each patient was collected on filter paper and transported to the Hospital Pediátrico David Bernardino molecular laboratory for identification of malaria parasites by PCR. The cytb-qPCR-method described by Xu et al. [14] was used, and *Plasmodium* species was confirmed by restriction fragment length polymorphism (RFLP) analysis of the PCR-amplified fragments.

### Variables

Malaria infection was defined as a positive PCR test result on any occasion after hospital admission. Severe malaria was defined, using all available data, according to the World Health Organization (WHO) guideline for research purposes [15], which requires one or more of the following findings: severely impaired level of consciousness (GCS score < 11), acidosis (base deficit > 8 meq/L or plasma bicarbonate < 14 mmol/L), hypoglycaemia (blood or plasma glucose < 2.2 mmol/L), severe malarial anaemia (Hb < 50 g/L together with a parasite count > 10 000/µL), renal impairment (plasma or serum creatinine > 265 µmol/L or blood urea > 20 mmol/L), jaundice (plasma or serum bilirubin > 50 mmol/L together with a parasite count > 100 000/µL), pulmonary oedema (radiologically confirmed or clinically diagnosed with oxygen saturation < 92%), significant bleeding (recurrent or prolonged bleeding from nose, gums or venipuncture sites, haematemesis or melaena), shock (prolonged capillary refill time, temperature gradient on leg or systolic blood pressure < 70 mmHg) or hyperparasitaemia (> 10%, corresponding to a parasite count > 500 000/µL) [16]. As thick film microscopy was not performed to all children, a parasite count was not required for the definition of severe malarial anaemia but taken into account if available. Pulmonary oedema was defined as dyspnea requiring oxygen treatment, if no other explanation for breathing difficulty could be identified. Analysis of the acid–base balance was not included the study and, therefore, the grade of acidosis was not evaluated as a sign of severe malaria.

Signs of raised intracranial pressure were defined as a bulging fontanel, vomiting, meningism, irritability, decreased consciousness or disorientation. In terms of disease outcomes, severe neurological sequelae were defined as blindness, quadriparesis or quadriplegia, severe psychomotor retardation, or hydrocephalus requiring a shunt; any neurological sequelae also comprised milder deficits such as ataxia, monoparesis and hemiparesis.

A decreased level of consciousness was defined as a GCS score < 15. Due to the lack of resources for performing ophthalmoscopy and the ambiguity associated with the clinical diagnosis of CM, this tool was not used in the analyses. Bacterial meningitis was defined similar to the WHO guidelines: if a child had compatible symptoms and signs, a CSF leucocyte count ≥ 100 cells x 10E6/L, or a CSF leucocyte count 10–100 cells x 10E6/L and either an elevated CSF protein concentration (> 100 mg/dL) or a decreased CSF glucose concentration (< 40 mg/dL), it was defined as a probable case of bacterial meningitis. A confirmed case required bacterial identification by culture or non-culture methods (Gram stain, antigen detection, PCR) [17].

The given treatment was retrospectively reviewed, acknowledging that the malaria PCR result was not available when the attending physician decided on treatment. However, routine microscopy of malaria parasites had been done as usual at the hospital. Children were treated according to the routine of the hospital, at the discretion of the attending physician. First-line treatment of severe malaria at the ward consisted of either quinine or artesunate.

### Statistical analysis

The characteristics of the cohort were presented using basic descriptive statistics. Potential differences in the clinical presentation and the disease course between children with and without malaria parasitaemia were assessed using the Pearson chi-square test (or the Fisher’s exact test in the case of low expected cell values in the 2 × 2 table) and the Mann–Whitney U test. A Kruskal–Wallis test was used to estimate the effect of the day of sampling on PCR test results, and multinomial logistic regression was used to assess the relationship of laboratory parameters and the odds of malaria parasitaemia. Statistical analyses were conducted using IBM SPSS Statistics software, version 27 (IBM Corp., NY, US) and the MedCalc software’s Diagnostic test evaluation calculator [18].

### Ethical aspect

Children were treated according to the routine of the hospital, regardless of study participation. Informed consent was obtained from all participants’ guardians. The study protocol was approved by the Ethics Committee of the Paediatric Hospital of Luanda on April 15, 2014.

## Results

### Malaria test results

**Fig. 1:**
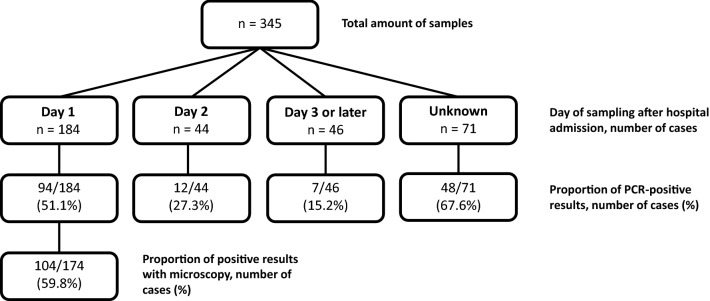
Collected samples for malaria PCR analysis

In total, 345 children (median age 7 years old, ranging from 1 month to 14 years old) were enrolled in the study. 161/345 children (46.7%) had a positive malaria PCR test result, while 184/257 (71.6%) had a positive microscopy result during their hospital stay. Figure 1 depicts when the samples for malaria PCR were collected after hospital admission, and the proportion of positive test results on each day of sampling. The proportion of positive test results differed according to the time of sampling (p < 0.001): the later the sample was obtained after hospital admission, the fewer were positive by PCR.

All positive cases were confirmed to be *P. falciparum.* 133 of the 161 (82.6%) parasitaemic children fulfilled the definition of severe malaria, according to the clinical and laboratory data available. The most common manifestation of severe malaria was a GCS score < 11 (n = 114), followed by dyspnea requiring oxygen supplementation (n = 61), and shock (n = 39). Commonly, these manifestations overlapped, and most children with severe malaria fulfilled more than one criterion (median number of fulfilled severity criteria was 2, ranging from 1 to 7).

Of the patients with available data, 82.6% (176/213) were documented to have received malaria treatment before PCR testing, either before admission or at the hospital emergency department before the sample for PCR analysis was taken. The percentage of positive PCR samples was lower among children receiving prior antimalarials compared to those not treated before sampling [63/176 (35.8%) vs. 21/37 (56.8%), p = 0.02].

### Clinical presentation

In general, children with malaria were older and attended school or day-care more frequently than those without parasitaemia (Table 1). Moreover, while only five out of 150 children with a positive malaria sample had a comorbidity, 29/178 (16%) of those without malaria parasitaemia had comorbidities, such as sickle cell anaemia, epilepsy, tuberculosis, or HIV infection, among others.

**Table 1:** Patient characteristics

Variables	Malaria-PCR positive	Malaria-PCR negative	P value^*^
Demography			
Total number of patients	161	184	N/A
Male sex (%)	88/161 (54.7%)	105/184 (57.1%)	0.65
Age, median years (IQR)	8 (5–10)	7 (4–10)	0.02
How many people in household, median (IQR)	6 (5–8)	6 (5–8)	0.73
Running water at home (%)	48/149 (32.2%)	56/178 (31.5%)	0.88
Electricity at home (%)	104/153 (68.0%)	130/180 (72.2%)	0.40
WC at home (%)	140/149 (94.0%)	164/177 (92.7%)	0.64
Attends school /daycare (%)	112/148 (75.7%)	98/173 (56.6%)	< 0.001
Comorbidity (%)	5/150 (3.3%)	29/178 (16.3%)	< 0.001
Pre-admission illness			
Duration of pre-admission illness, median days (IQR)	4 (3–6)	5 (3–10)	0.001
Fever at home (%)	95/154 (61.7%)	107/181 (59.1%)	0.63
Vomiting at home (%)	70/154 (45.5%)	85/181 (47.0%)	0.78
Convulsions at home (%)	92/141 (65.2%)	138/175 (78.9%)	0.007
Focal convulsions at home (%)	18/125 (14.4%)	18/166 (10.8%)	0.36

Data are presented as number of cases and percentages, unless otherwise specified

*IQR* interquartile range

^*^P values were obtained using a Pearson’s chi-square test or the Mann–Whitney U test

Children with a positive malaria PCR result presented to hospital with a shorter duration of illness (median duration of four vs. five days). In addition, a higher frequency of children without malaria suffered from convulsions before hospital admission, compared to those with documented malaria (Table 1).

At the emergency department, signs of malnutrition or an infection outside the central nervous system were more common in malaria-negative patients (Table 2). Overall, the children were severely ill, presenting with a median GCS score of 8 in both groups, and several signs of raised intracranial pressure. Children with malaria had more profound anaemia and thrombocytopenia compared to malaria-negative children. In addition, those with malaria parasitaemia had a higher CRP level than those without (Table 2). If a child presented with a haemoglobin level under 8 g/L, a thrombocyte count under 200 × 10E9/L and a CRP level over 100 mg/L, the odds of a positive malaria PCR sample was 4.8-fold (95% confidence interval [CI], 2.2–10.4) compared to children not fulfilling these criteria.

**Table 2:** Presentation at emergency department

Variables	Malaria-PCR positive	Malaria-PCR negative	P value^*^
Clinical findings			
GCS score on admission	8 (7–9)	8 (7–9)	0.82
Number of signs of raised ICP	2 (2–3)	2 (2–3)	0.12
Convulsions at ED (%)	88/149 (59.1%)	113/180 (62.8%)	0.49
Capillary refill, seconds	2 (2–3)	3 (2–3)	0.10
Focal neurological deficits (%)	11/132 (8.3%)	21/160 (13.1%)	0.19
Infection focus outside CNS (%)	8/134 (6.0%)	32/161 (19.9%)	< 0.001
Clinically dehydrated (%)	36/136 (26.5%)	59/163 (36.2%)	0.07
Clinically malnourished (%)	28/138 (20.3%)	52/165 (31.5%)	0.03
Dyspnea at ED (%)	94/136 (69.1%)	117/163 (71.8%)	0.62
Blood test results^†^			
Haemoglobin, g/L	62 (51–81)	82 (63–103)	< 0.001
Leukocyte count, x 10E9/L	11.9 (7.6–15.5)	11.0 (7.6–16.3)	0.54
Polymorphonuclear leukocyte count, x 10E9/L	6.6 (4.6–9.8)	6.9 (4.2–9.8)	0.92
Thrombocyte count, x 10E9/L	73 (41–166)	207 (95–360)	< 0.001
CRP, mg/L	> 160 (150– > 160)	150 (55- > 160)	< 0.001
Glucose level, mmol/l	4.6 (4.2–5.9)	5.2 (4.2–6.7)	0.04
CSF test results			
Leukocyte count, x 10E6/L	0 (0–2)	0 (0–2)	0.26
Protein level, mg/dL	60.7 (31.6–99.4)	92.6 (48.8–157.6)	< 0.001
Glucose level, mmol/L	3.8 (2.7–4.6)	3.0 (1.7–4.4)	0.02

Data are presented as median values with interquartile range, unless otherwise specified

*CNS* central nervous system; *CRP* C-reactive protein; *CSF* cerebrospinal fluid; *ED* emergency department; *GCS* Glasgow coma scale; *ICP* intracranial pressure

^*^P values were obtained using a Pearson’s chi-square test or the Mann–Whitney U test

^†^Lowest haemoglobin and glucose level and highest leukocyte, polymorphonuclear leukocyte, and thrombocyte count and highest CRP value on days 1–2

In terms of the CSF findings, most of the children showed no pleocytosis. The CSF protein level was slightly elevated in both groups, although higher in malaria-negative cases. Moreover, the CSF glucose level was lower in children without malaria (Table 2). In total, 21 children had confirmed or probable bacterial meningitis, four of which simultaneously had a positive malaria PCR result. Bacterial meningitis was more frequent in children without malaria compared to those with a positive malaria PCR result (17/184 vs. 4/161, respectively; p = 0.009).

### Treatment and outcomes

In general, children with malaria received more blood transfusions and malaria treatment, but less antibiotics, anticonvulsants, corticosteroids, acyclovir, or tuberculosis treatment (Table 3). However, more than 90% of children with a negative malaria PCR sample received treatment for malaria, while 65% of children with malaria received antibiotics (Table 3). Children without malaria were hospitalized for a longer period than those with malaria (median length of hospitalization was 8 days vs. 6 days, respectively).

**Table 3:** Treatment and outcomes

Variables	Malaria-PCR positive	Malaria-PCR negative	P value*
In-hospital treatment			
Blood transfusion (%)	89/130 (68.5%)	64/159 (40.3%)	< 0.001
Oxygen treatment (%)	116/135 (85.9%)	140/163 (85.9%)	0.99
Malaria treatment (%)	135/139 (97.1%)	146/160 (91.3%)	0.03
Quinine treatment (%)	75/137 (54.7%)	101/161 (62.7%)	0.16
Anti-convulsives (%)	77/127 (60.6%)	115/156 (73.7%)	0.02
Antibiotics (%)	86/132 (65.2%)	134/160 (83.8%)	< 0.001
Corticosteroids (%)	7/121 (5.8%)	36/155 (23.2%)	< 0.001
Acyclovir (%)	6/121 (5.0%)	32/155 (20.6%)	< 0.001
TB treatment (%)	0/136 (0.0%)	13/168 (7.7%)	< 0.001
Length of hospitalization, median days (IQR)	6 (4–9)	8 (5–13)	< 0.001
Disease outcomes			
Death	37/148 (25%)	54/171 (31.6%)	0.19
Day of death, median (IQR)	3 (2–4)	5 (3–9)	< 0.001
SeNeSe at discharge	3/102 (2.9%)	17/104 (16.3%)	0.001
AnNeSe at discharge	5/97 (5.2%)	30/102 (29.4%)	< 0.001
Death or SeNeSe	40/139 (28.8%)	71/158 (44.9%)	0.004

Data are presented as number of cases and percentages, unless otherwise specified

*AnNeSe* any neurological sequelae; *IQR* interquartile range; *SeNeSe* severe neurological sequelae; *TB* tuberculosis

^*^P values were obtained with Pearson’s chi-square test or the Mann–Whitney U test

In total, 28.5% (91/319) of the children died. The case fatality ratio did not significantly differ between those with malaria and those without (37/148 and 54/171, respectively; p = 0.19), but children with malaria died closer to hospital admission (Table 3). When neurological deficits were also considered, a positive malaria sample was associated with fewer neurological sequelae in survivors and a better composite outcome (including death or severe neurological sequelae) (Table 3).

In parasitaemic children, potential associations between certain WHO-defined severity criteria for malaria and the survival were investigated [19]. The case fatality ratio did not differ between parasitaemic children with or without hypoglycemia (p = 0.50), pulmonary oedema (p = 0.37), shock (p = 0.49), or a GCS-score below 11 (p = 0.91) or below 8 (p = 0.44). However, renal impairment increased the odds of death fivefold (95% CI 1.1–22.7, p = 0.04). In terms of anti-malarial treatment, no differences in survival were noted between parasitaemic children receiving quinine versus artemisinin derivatives [47/61 (77.0%) vs. 50/64 (78.1%), respectively, p = 0.89].

### Comparison of microscopy and cytb-qPCR

The performance of routine microscopy against the cytb-qPCR-method was assessed in children with both routine thick film microscopy and PCR analysis performed from samples obtained on admission (n = 174). Using the PCR as the reference method, microscopy yielded a sensitivity of 96.8% (95% CI 90.1–99.3) and a specificity of 82.7% (95% CI 72.7–90.2). This corresponded to a positive predictive value of 86.5% (95% CI 80.0–91.2) and a negative predictive value of 95.7% (95% CI 88.0–98.6) (Additional file 1).

Thus, 14/104 (13.5%) of the children with a positive microscopy result were PCR-negative, and 3/93 (3.2%) of the children with a negative microscopy result had malaria parasites in the blood detected by PCR. Within the PCR-negative group, children with a positive microscopy differed in terms of a lower haemoglobin level (median haemoglobin of 76 g/L vs. 91 g/L, p = 0.04), while no significant differences were noted in other laboratory parameters on admission or the survival. As for the three PCR-positive children with a negative microscopy, one had culture-confirmed pneumococcal meningitis, but no differences in the blood test results emerged. Two of these children died, resulting in a higher case fatality ratio than in children with both positive microscopy and positive PCR test result (p = 0.04).

## Discussion

In this cohort of children presenting to hospital with a decreased level of consciousness, half (161/345, 46.7%) had PCR-confirmed malaria parasitaemia. Few clinical findings on admission differentiated children with malaria from those without, although differences in laboratory findings clearly emerged. The case fatality ratio did not significantly differ between parasitaemic and non-parasitaemic children.

Children with malaria were slightly older than those without malaria and attended school or day-care more often (Table 1). Overall, the cohort comprised older children than previous reports of childhood non-traumatic coma; however, the inclusion of children also with less severely impaired consciousness might contribute to this finding [20].

In terms of laboratory parameters, children with malaria were more frequently anaemic and thrombocytopenic on admission and showed higher serum levels of CRP compared to those without parasitaemia (Table 3). In line with this, CRP has previously been shown to associate with the degree of parasitaemia, as well as other markers of severe malaria [21].

Children with malaria died earlier after hospital admission than children without. Indeed, death due to severe malaria typically occurs soon after hospital admission, the majority within 24 h [3, 22–24]. The case fatality rate of parasitaemic children in this cohort was 25%, slightly higher than previously reported for CM in African children [25]. Although malaria-positive children presented to hospital earlier during the illness than children without malaria (the median duration of pre-admission illness was 4 vs. 5 days, respectively), their hospital admission occurred later than commonly reported for children with CM, which might partly explain for the poor prognosis [4, 22, 25].

On the other hand, the incidence of neurological sequelae (Table 3) remained similar or even lower than previously reported for CM [22, 23, 25]. Overall, a positive malaria-PCR test result related to a better outcome when also neurological deficits were considered (Table 3). This probably reflects the lower risk of neurological sequelae after CM compared to its differential diagnoses such as bacterial meningitis. In fact, the composite outcome (death or severe neurological sequelae) of malaria-negative children in this report is comparable to the outcome of children with bacterial meningitis noted in a previous study at the same institution [26]. Similar findings, suggesting that malaria in comatose children associates with a better prognosis compared to differential aetiologies, have been previously reported [20].

The given treatment—which often included both anti-malarials and antibiotics—is in line with the WHO guideline for treatment of severe malaria [27]: In endemic regions, severely ill children should be treated initially with both anti-malarials and empirical antibiotics, until an accurate diagnosis has been established. Quinine was broadly used at the time this study was conducted, although the WHO currently recommends artemisinin derivatives as the first-line treatment [27, 28].

It remains unclear how many of these children with malaria had an impaired consciousness due to severe malaria. Of the 161 children with a positive malaria PCR result, only two had culture-confirmed bacterial meningitis. However, 17% of them received antibiotic treatment prior to hospital admission. Profound anaemia and thrombocytopenia were more common in malaria-positive children, which might point towards the parasitaemia being significant.

There is also uncertainty about the number of children with CM, as severe anaemia or acidosis, hypoglycaemia, post-ictal status or malaria-associated acute kidney injury and subsequent uraemia may also impair consciousness. Among the 161 children with malaria parasitaemia, 42 had other identified possible causes of decreased consciousness (severe anaemia, hypoglycaemia, uraemia and probable or confirmed bacterial meningitis, among others), leaving 119 children with strictly defined CM. Nonetheless, as different signs of severe falciparum malaria commonly co-existed, identification of one sign does obviously not exclude another [3]. Unfortunately, the presence of malarial retinopathy was not investigated during this study.

The performance of thick film microscopy against PCR yielded a sensitivity of 96.8% and a specificity of 82.7%. Compared to previous reports, which have demonstrated a highly variable performance, thick film microscopy at Hospital Pediátrico David Bernardino in Luanda seemed to perform reasonably well [9, 11, 29, 30].

The findings of this study must be interpreted in light of potential limitations. The sample for the malaria PCR analysis was taken on the third day or later in 13% of children, and the exact date of sampling was unknown in 21%. Although not probable, it cannot be excluded that some of the children with parasitaemia acquired the infection in hospital, which would lead to an overestimation of the baseline incidence of malaria. It is likewise possible that some of the negative samples taken later during the treatment were positive on admission, leading to misclassification of these patients.

Some data were also missing for the definition of severe malaria according to the WHO, such as serum bilirubin levels and severity of acidosis. These data would have been of interest, especially while addressing the proportion of CM. Moreover, also information on the treatment given before hospital admission was limited in some patients. Finally, the true incidence of decreased consciousness due to severe malaria (including CM) in this cohort remains uncertain.

## Conclusions

In Luanda, approximately half of the children presenting to hospital with a decreased level of consciousness have malaria parasitaemia. Few clinical characteristics aid in differentiating children with parasitaemia from those without, but blood count and CRP analyses might be helpful in assessing the risk of malaria infection. Targeted thick film microscopy on admission remains an adequate tool for diagnosing malaria in this population.

Despite that most of these severely ill children received both antibiotic and antimalarial treatment, 28.5% still died. Although the case fatality ratio did not significantly differ between parasitaemic children and those without parasitaemia, a positive malaria PCR result was associated with a better prognosis overall. Nonetheless, regardless of etiology, children presenting to hospital with an impaired consciousness in these circumstances require meticulous attention in order to optimize the given treatment and improve the outcome.

## Supplementary Information

**Additional file 1: Table S1.** Performance of microscopy against the cytb-qPCR method. Description: A table showing the positive and negative results of the cytb-qPCR method and thick film microscopy from samples obtained on admission.

## Data Availability

The data that support the findings of this study are available from the corresponding author (O.S.) on reasonable request. The data are not publicly available due to privacy and ethical concerns.

## Abbreviations

CM: Cerebral malaria
GCS: Glasgow Coma Scale
CNS: Central nervous system
CRP: C-reactive protein
CSF: Cerebrospinal fluid
RFLP: Restriction fragment length polymorphism
WHO: World Health Organization
IQR: Interquartile range
ED: Emergency department
ICP: Intracranial pressure
AnNeSe: Any neurological sequelae
SeNeSe: Severe neurological sequelae
TB: Tuberculosis
