# Supplementary material for: PCR-confirmed malaria among children presenting with a decreased level of consciousness in Angola: a prospective, observational study
Source: Malar J. 2023 Apr 22;22:130. doi: 10.1186/s12936-023-04556-9 (PMC10122800; doi:10.1186/s12936-023-04556-9)
Supplement: Supplementary file 1 — Additional file 1: Table S1. Performance of microscopy against the cytb-qPCR method. Description: A table showing the positive and negative results of the cytb-qPCR method and thick film microscopy from samples obtained on admission. [file 12936_2023_4556_MOESM1_ESM.docx]

| **Additional file 1.** Performance of microscopy against the cytb-qPCR method | | | |
| --- | --- | --- | --- |
|  | **Positive by PCR** | **Negative by PCR** | **Total** |
| Positive by microscopy | 90 | 14 | 104 |
| Negative by microscopy | 3 | 67 | 70 |
| Total | 93 | 81 |  |
| Data are presented as number of cases, including only children with both microscopy and PCR performed from samples obtained on admission. | | | |
